# A Mobile App Specifically Designed to Facilitate Exercise in Parkinson Disease: Single-Cohort Pilot Study on Feasibility, Safety, and Signal of Efficacy

**DOI:** 10.2196/18985

**Published:** 2020-10-05

**Authors:** Merrill R Landers, Terry D Ellis

**Affiliations:** 1 Department of Physical Therapy School of Integrative Health Sciences University of Nevada, Las Vegas Las Vegas, NV United States; 2 Department of Physical Therapy and Athletic Training College of Health & Rehabilitation Sciences: Sargent College Boston University Boston, MA United States

**Keywords:** Parkinson disease, smartphone, mobile phone, telehealth, telerehabilitation, digital health, physical therapy

## Abstract

**Background:**

Many people with Parkinson disease do not have access to exercise programs that are specifically tailored to their needs and capabilities. This mobile app allows people with Parkinson disease to access Parkinson disease–specific exercises that are individually tailored using in-app demographic questions and performance tests which are fed into an algorithm which in turn produces a video-guided exercise program.

**Objective:**

To test the feasibility, safety, and signal of efficacy of a mobile app that facilitates exercise for people with Parkinson disease.

**Methods:**

A prospective, single-cohort design of people with Parkinson disease who had downloaded the 9zest app for exercise was used for this 12-week pilot study. Participants, who were recruited online, were encouraged to exercise with the full automated app for ≥150 minutes each week. The primary endpoints were feasibility (app usage and usability questions) and safety (adverse events and falls). The primary endpoints for signal of efficacy were a comparison of the in-app baseline and 8-week outcomes on the 30-second Sit-To-Stand (STS) test, Timed Up and Go (TUG) test, and the Parkinson’s Disease Questionnaire 8 (PDQ8).

**Results:**

For feasibility, of the 28 participants that completed the study, 12 participants averaged >150 minutes of app usage per week (3 averaged 120-150, 4 averaged 90-120, and 9 averaged less than 90 minutes). A majority of participants (>74%) felt the exercise was of value (16/19; 9 nonrespondents), provided adequate instruction (14/19; 9 nonrespondents), and was appropriate for level of function (16/19; 9 nonrespondents). For safety, there were no serious adverse events that occurred during the app-guided exercise. There were 4 reports of strain/sprain injuries while using the app among 3 participants, none of which necessitated medical attention. For signal of efficacy, there was improvement for each of the primary endpoints: STS (*P*=.01), TUG (*P*<.001), and PDQ8 (*P*=.01).

**Conclusions:**

Independent, video-guided exercise using a mobile app designed for exercise in Parkinson disease was safe and feasible though there was variability in app usage. Despite this, the results provide evidence for a signal of efficacy as there were improvements in 3 of the 3 outcomes.

**Trial Registration:**

ClinicalTrials.gov NCT03459586; https://clinicaltrials.gov/ct2/show/NCT03459586

## Introduction

Exercise is an important therapy for people with Parkinson disease. It has a positive effect on physical capacity and physical/cognitive function, including improvements in gait, mobility, posture, and balance [1]. In addition, several meta-analyses and systematic reviews have concluded that exercise and physical therapy improve many Parkinson disease–specific motor and nonmotor symptoms [2-6]. Importantly, there are several lines of evidence, using rodent Parkinson disease models and in humans with Parkinson disease, that suggest a possible disease-modifying effect of exercise on Parkinson disease [7-22].

Despite the evidence revealing the benefits of exercise in Parkinson disease, many people with Parkinson disease do not participate regularly in exercise and the reasons for this are complex [23]. First, people with Parkinson disease have many of the same type of inherent intrinsic and extrinsic barriers that are present in healthy, older adults. These include a lack of motivation, finances, knowledge, skill, accessibility to exercise facilities, and transportation, among other things [24-26]. Many of these issues are compounded in Parkinson disease because of Parkinson disease–specific nonmotor symptoms, including fatigue [27] and depression [28]. In addition, low outcome expectation, lack of time, and fear of falling have been identified as important perceived barriers to exercise in people with Parkinson disease [29].

While exercise is not routinely recommended by neurologists in the early course of Parkinson disease treatment [30], neurologists are frequently the first to introduce the importance of exercise to people with Parkinson disease. Typically, the patient is referred to a physical therapist who will prescribe an exercise program that is scaled to the person’s health and functional status. Ideally, people with Parkinson disease will continue to exercise independently or in a community program after they have finished their therapy and then return to their physical therapist every 6-12 months to recalibrate the exercise program to meet the challenges of new and increasing impairments brought on by disease progression. Unfortunately, many have difficulty sustaining regular engagement in an exercise program between physical therapy visits [24,31]. Therefore, a relatively large proportion fall back into previous habits and discontinue their exercise after discharge from supervised therapy. Subsequently, gained benefits are lost and prospective benefits of continuing exercise therapy are unrealized [32].

One solution that addresses many of the aforementioned barriers to sustained exercise participation is a recently developed, commercially available mobile app (9zest Parkinson’s Therapy [33], a subsidiary of Moterum Technologies [34]), which was developed by a software engineer with Parkinson disease. This app uses self-report questions and app-guided performance tests to assess status at baseline and progress the exercise program over time. Using these self-report and performance metrics, the app constructs a customized exercise program using a proprietary algorithm. The app selects exercises from a library of exercise videos specifically designed for people with Parkinson disease and is calibrated to one’s current level of function. The 9zest app also includes several behavior change techniques, including prompts/cues, goal setting, graded tasks, and performance feedback. Currently, there are a limited number of apps reported in the literature that promote exercise or physical activity for people with Parkinson disease [35,36].

The 9zest app specifically aims to mitigate many of the barriers to exercise participation. First, the app can be used in the privacy of one’s home. Because prominent barriers to participation include lack of accessibility and limited time, this app allows those with transportation problems or those in rural areas and “medical/exercise deserts” to have access, at a convenient time, to a semicustomized exercise program for people with Parkinson disease. In addition, because it has periodic assessments, it helps improve participant motivation through goal setting and feedback [29]. Lastly, because the app is relatively low cost, it is ideal for financially disadvantaged populations. While the 9zest app appears promising, it has not been evaluated scientifically to determine its utility for people with Parkinson disease. Therefore, Aim 1 was to test the feasibility (adherence and user feedback) of using the app for independent exercise over 12 weeks for people with Parkinson disease. Aim 2 was to test the safety (adverse events and falls) and Aim 3 was to detect a signal of efficacy for lower extremity strength, functional gait, and quality of life in people with Parkinson disease. Identifying signals of efficacy is important in early studies, such as that described here, as they allow inference about proof of concept and whether continued scientific exploration using more rigorous clinical trials is warranted [37].

## Methods

### Study Design

A prospective, single-cohort design was implemented for this pilot study wherein participants who had downloaded the commercially available 9zest app were invited to participate in this study via an in-app message. Based on responses to the inclusion and exclusion criteria during in-app consenting, qualified participants were invited to complete the in-app baseline assessment (detailed later). These measures were used by the app to construct an individualized 12-week exercise program. All study-related tasks were conducted without direct contact from a member of the research team. Participants were instructed to be tested in the “on” Parkinson disease medication state for testing and app-guided exercise. For Aim 1 (feasibility), participation data (minutes of use) were recorded by the app and analyzed over the 12-week intervention period. Additionally, participants were asked questions about the usability of the app at the conclusion of the 12-week study. For Aim 2 (safety), adverse events data were tracked via an in-app question every 2 weeks of the 12-week study. Fall data were tracked via an in-app question after every exercise session. Outcomes for Aim 3 (signal of efficacy) were assessed at baseline, 8 weeks, and 12 weeks. However, the primary endpoint for the signal of efficacy was the 8-week measurement point as it was thought that waning adherence or more drop outs would occur over time; therefore, 8 weeks was chosen to optimize the ability to detect a signal of efficacy. The 12-week measurement was secondary.

### Participants

People with Parkinson disease who had downloaded the commercially available app were automatically invited to participate in the study. Those meeting all inclusion criteria (English speaking, between 40 and 75 years old, self-report neurologist-diagnosed Parkinson disease, willingness to participate in a 12-week study, able to stand unassisted for 10 minutes, and stable on Parkinson disease medications and deep brain stimulation for 3 months prior to participation) and not having any of the exclusion criteria (diagnosed with dementia; comorbidities that would preclude exercise participation or increase participant risk [eg, severe osteoarthritis/pain, stroke, severe respiratory problems, traumatic brain injury, neuromuscular disease, atrial fibrillation, poorly controlled cardiovascular disease, limb amputation, osteoporosis]; vision or hearing impairment that would interfere with app use; fall that required physician evaluation [emergency visit, urgent care, or hospitalization] within the past year; use of an assistive device for walking; and currently exercising more than 60 minutes per week on average) were invited to advance to consent using an online form. This study was approved by the University of Nevada, Las Vegas Institutional Review Board. Participants were recruited online from a sample of convenience from July 2018 to May 2019, clinical trial registry (clinicaltrials.gov [NCT03459586]) using social media advertisements, health care provider referrals, and snowball recruitment strategies wherein participants were encouraged to recruit other participants among their acquaintances.

### Sample Size Calculation

The sample size was estimated using Aim 3 and was calculated with the “paired *t* tests using effect size module” in PASS 19.0 [38] (NCSS, LLC). The sample size estimation was based on an effect size between 0.39 and 0.52 for the 30-second Sit-To-Stand (STS) test from another exercise study for people with Parkinson disease [39]. The analysis was based on a one-sided test at 80% power with the significance level at .05 and a 15% dropout rate; this estimation was conservatively based on the same exercise study for people with Parkinson disease that had an 11.1% dropout rate [39]. With dropouts, a sample size range between 30 (effect size=0.52) and 53 participants (effect size=0.39) was estimated.

### Outcomes

Primary and secondary endpoints were all assessed via the app. Principal analyses took place at the 8-week point and exploratory analyses took place at the 12-week point. The primary endpoint of feasibility (Aim 1) was assessed by in-app tracking of app exercise in minutes. Usability was assessed using the following statements at the conclusion of the 12-week study with a 5-point Likert scale (5=strongly agree, 4=agree, 3=neutral, 2=disagree, and 1=strongly disagree): (1) *I believe this activity could be of some value to me*; (2) *I thought the app provided adequate instruction on the exercises*; (3) *I enjoyed using the app to help me exercise*; (4) *I would recommend this app to others with Parkinson disease*; and (5) *The exercises were appropriate for my level of functioning*.

The primary endpoint of safety (Aim 2) was assessed by tracking adverse events (assessed every 2 weeks over 12 weeks with a short in-app question asking if they have experienced any app exercise–related adverse events). Participants were asked questions about the occurrence of the following while doing the app-guided exercises: strains/sprains, chest pain, shortness of breath, and dizziness. If an event was reported, participants were asked follow-up questions to record the details (ie, pain rating, if it required medical attention, outcome). Safety was also measured by asking about the occurrence of a fall/s and other medical issues/events that occurred during the exercise (assessed after every app exercise session using an in-app question). In addition, at the conclusion of the 12-week study participants were asked the following statement with a 5-point Likert scale (strongly agree, agree, neutral, disagree, and strongly disagree): *I felt safe doing the exercises using the app*.

The following endpoints for Aim 3 (signal of efficacy) were assessed at baseline, and at the 8-week (primary) and 12-week (secondary) measurement points as part of the in-app assessment:

- 30-second STS, which is a measure of functional lower extremity strength with good reliability in people with Parkinson disease [40,41] and a minimal detectable change (MDC) of 3 [40];
- Timed Up and Go (TUG), which is a test of mobility and dynamic balance with evidence for good reliability in people with Parkinson disease [42-44] and an MDC of 4.85 [45]; and
- Parkinson’s Disease Questionnaire 8 (PDQ8), which is a shortened version of the PDQ39, a Parkinson disease–specific quality of life measure, which has good reliability in people with Parkinson disease [46-49] and an MDC of 5.43 [45].

**Figure 1:**
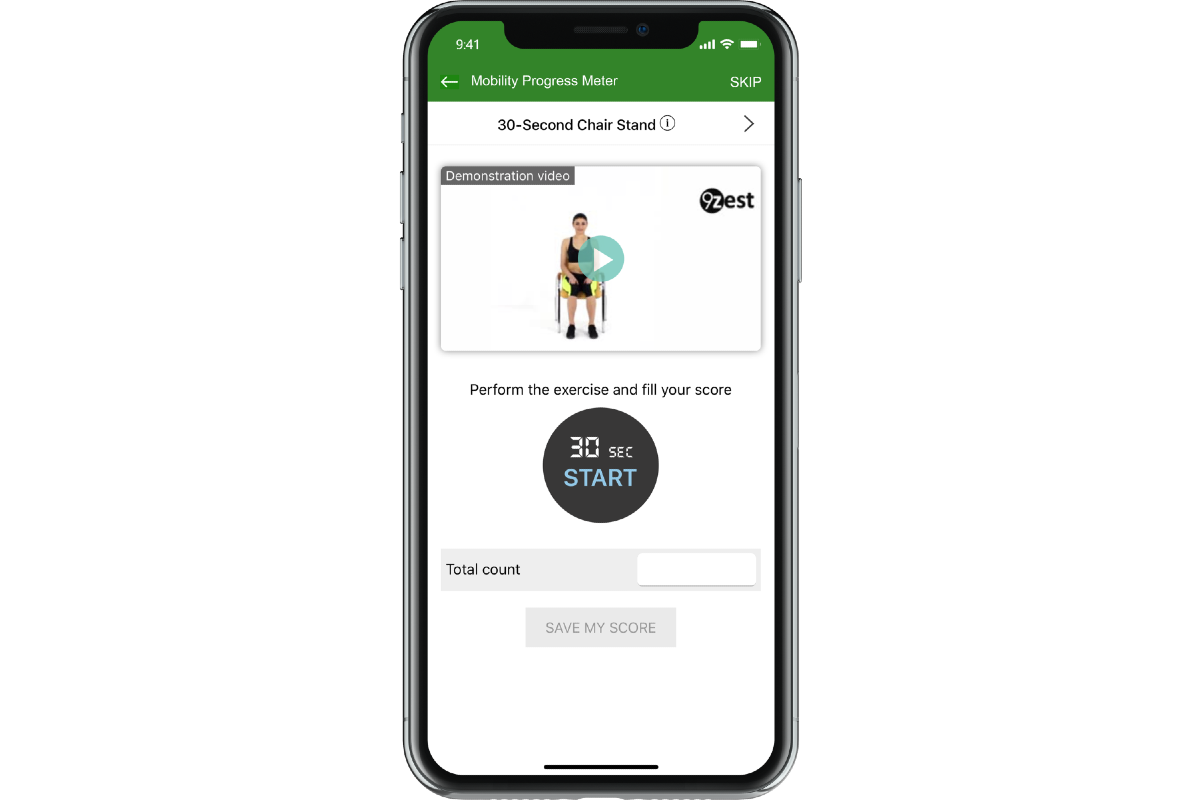
An example (30-second Sit-To-Stand test) of the in-app performance-based assessment used as a primary endpoint for the study and also used by the 9zest Smart to construct the exercise routine.

The STS and TUG tests were preceded by a demonstration video and an explanation of the test prior to the assessment using a built-in timer (Figure 1). In addition to the STS, TUG, and PDQ8, the Global Rating of Change score was asked at the conclusion of the 12-week study.

### Exercise Intervention

**Figure 2:**
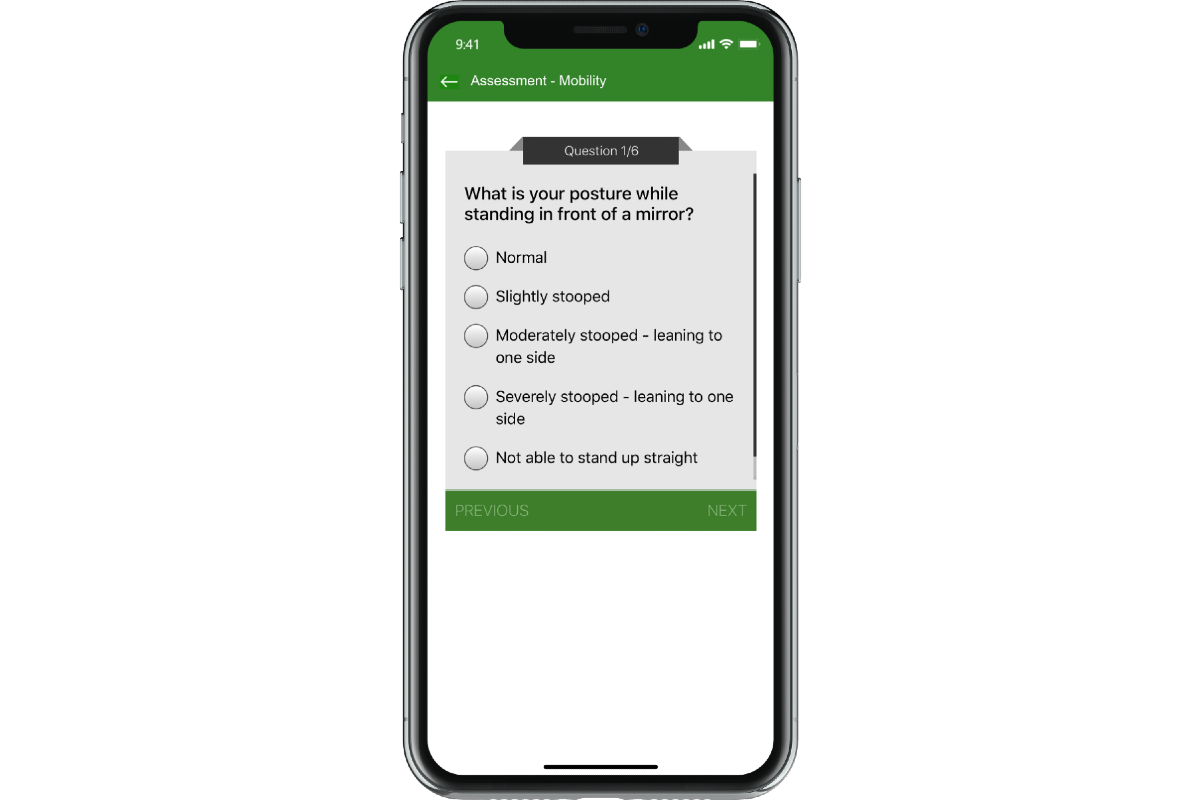
An example of an in-app self-report question that is used in the algorithm to construct the exercise program.

**Figure 3:**
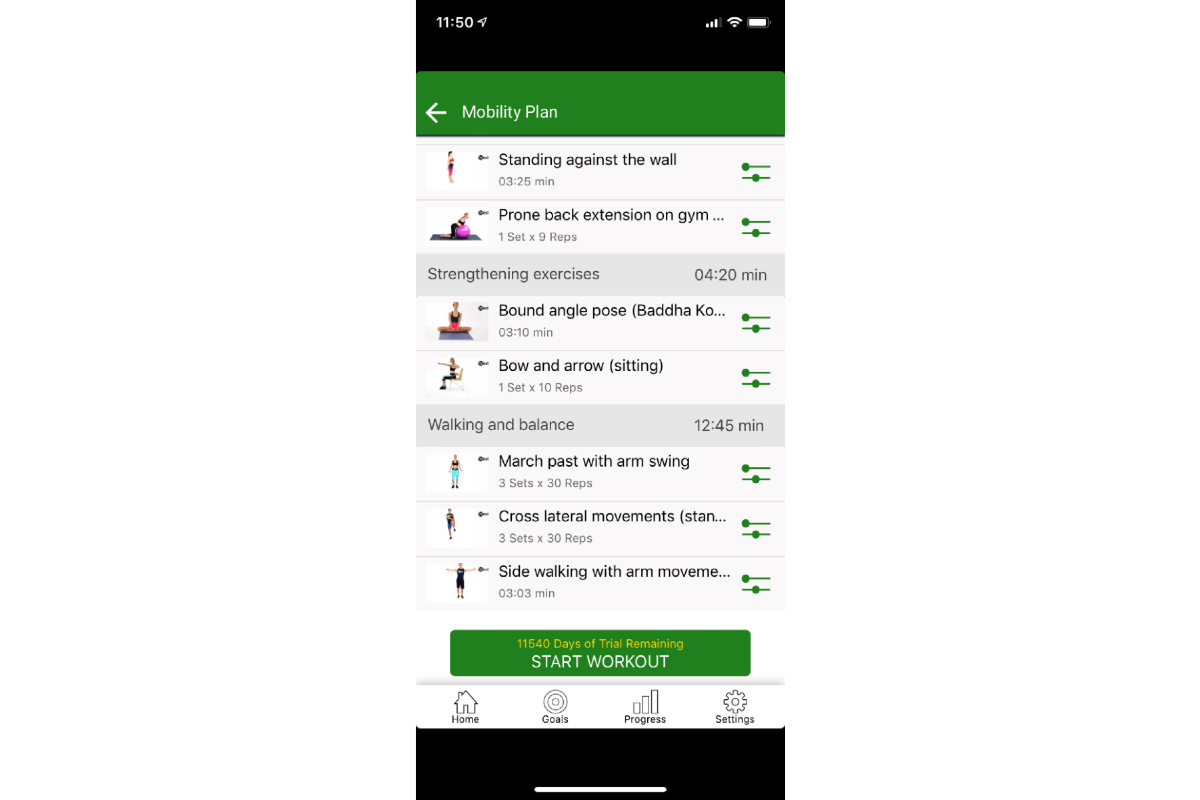
An example of an exercise program.

**Figure 4:**
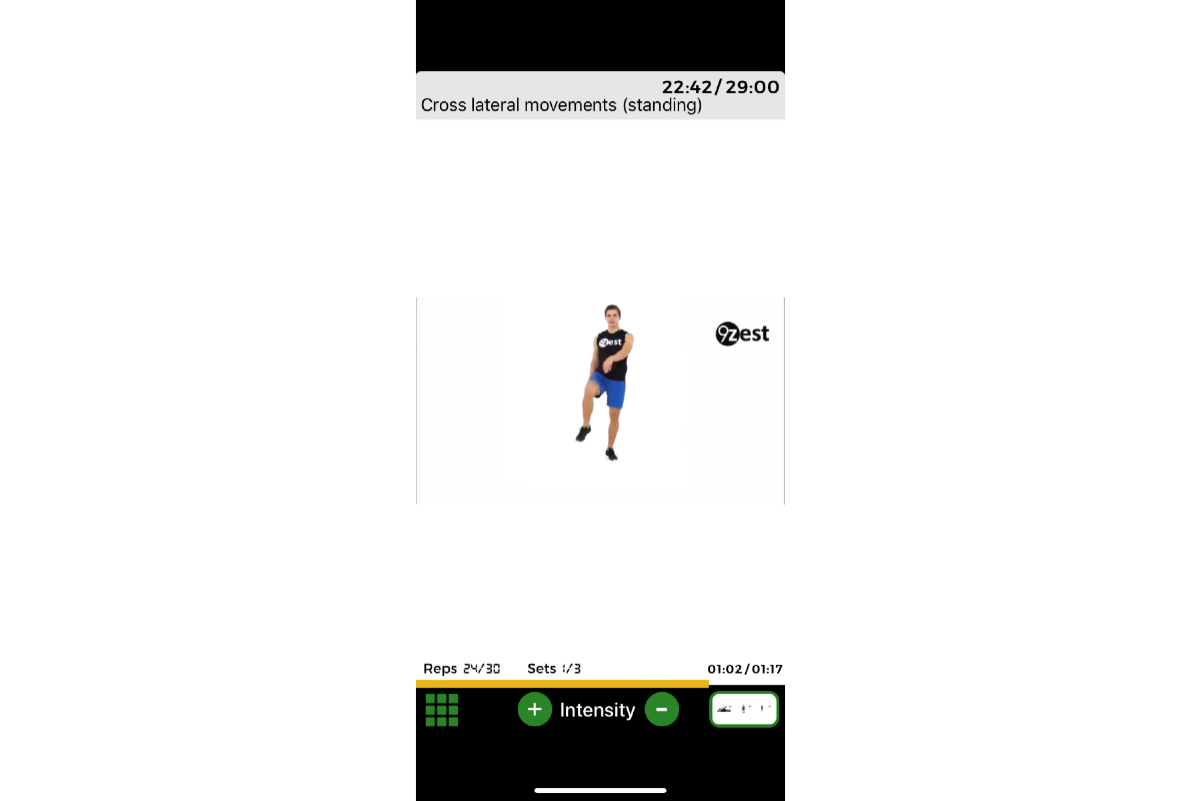
An example of a specific app-guided exercise (b).

Participants were encouraged to use the app on their phone or tablet for at least 150 minutes per week (eg, 3-5 days per week at 30-60 minutes each session) for 12 weeks. After registering on the app and completing several self-report Likert scale questions (ie, standing posture, tremors, balance, fall history, turning in bed, body movements with activities of daily living; Figure 2) and performance-based assessments (ie, STS, timed 360 degree turn, TUG, timed shirt removal), the 9zest app, using a proprietary algorithm (9zest Smart), constructed an exercise regimen for the participant’s level of function. The customized exercise regimen was constructed from a library of over 1000 original exercise videos, developed by physical therapists. The library consisted of exercise in the following categories: aerobic, strengthening, balance, yoga based, range of motion/stretching, meditation based, and speech therapy exercises. However, because mobility was assigned as the in-app goal for each participant, the 9zest Smart algorithm drew exercises from the strengthening, balance, and stretching categories. The 9zest Smart, the app’s intelligent engine, determined the regimen and levels of exercises for a user based on the most recent assessment (Figures 3 and 4). Thus, the app chooses the exercise program based on the primary goal (ie, mobility) and then uses the responses from self-report questions and the data from performance-based tests to determine the severity of the Parkinson disease. From this information, the app selects exercises that are consistent with the primary goal and at the appropriate level of function based on one’s severity of Parkinson disease. At preset intervals (generally after 2 weeks), the app reassessed functional capacity using the same self-report questions and performance-based measures. The 9zest Smart algorithm adjusted the type, duration, and intensity of each exercise in the constructed exercise regimen. All of the exercises and dosing features are consistent with contemporary, clinical-based physical therapy practice as determined by the physical therapy development team who helped build the exercise library.

**Figure 5:**
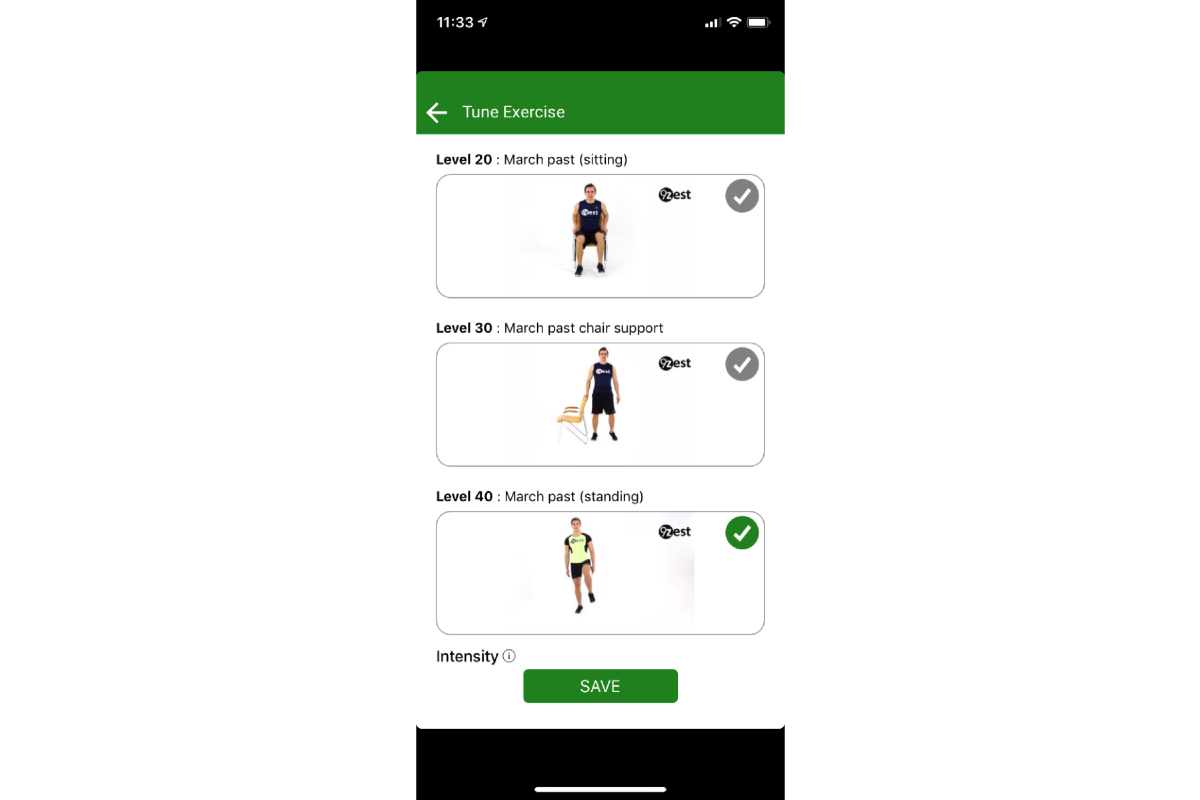
An example of graded exercises based on level of function.

Because app-guided exercises are typically performed independently, safety was a primary concern in the development. All exercises in the library were deemed safe for people with Parkinson disease by the physical therapy development team for level of impairment and function. However, because this had not been scientifically vetted, it became a primary aim of this study. An additional safety feature was an audiovisual demonstration of each exercise to ensure that the exercise was performed with the proper technique. After the demonstration, the participant would then follow along with the audiovisual of the exercise in real time (Figures 3 and 4). If participants were deemed a fall risk by the in-app assessment, then all balance exercises would be scaled to level of function. For example, the balance exercise may be performed in sitting or at a counter/chair with hand contact (Figure 5). The exercise library consists of many variations of the same exercise (eg, standing, standing with support, sitting and lying down) and the decision on which exercise is selected for the exercise regimen is determined by the in-app assessment. Thus, the 9zest Smart algorithm would select an appropriate exercise variation with the intent of minimizing the risk for an injury or a fall. Additionally, before the session started, a warning message was displayed asking the participant to skip any exercises that might cause any pain or imbalance. Lastly, the in-app instruction reminded participants to work within their own limits to avoid any injury or fall.

### Data Analysis

Data were analyzed using SPSS version 24.0 (IBM SPSS Statistics for Windows) and α=.05 (where α is a priori level of significance). For Aim 1 (feasibility), basic descriptive statistics were used for in-app tracked exercise minutes and participants’ responses to Likert scale questions. For Aim 2 (safety), basic descriptive statistics were used for the number of adverse events, falls, and a Likert-style question on whether the app was safe. For Aim 3 (signal of efficacy), the primary endpoint of the pre and 8-week outcome measures were analyzed using paired *t* tests to determine if app-guided exercise improved outcomes over the first 8 weeks of the study. In addition, the number of participants who improved beyond the MDC was tabulated for each of the primary endpoints. For the secondary endpoint of the 12-week outcomes and to determine if improvement continued after the 8-week measurement point, a one-way repeated measures ANOVA was used to compare the pre, 8-week, and 12-week outcomes. Descriptive statistics were used to report the results of the Global Rating of Change responses. To determine if there was a dosing effect, those who used the app as instructed (≥150 minutes per week) were compared with those who did not using a 2 (≥150 minute average per week: yes or no) × 2 (time: premeasurement, 8-week measurement) mixed factorial ANOVA. To explore this further, Pearson correlational coefficient analyses were performed to determine if the minutes of app use was associated with overall improvement (8-week measurement – premeasurement) for each of the 3 primary endpoints (ie, STS, TUG, and PDQ8).

## Results

### Recruitment

**Figure 6:**
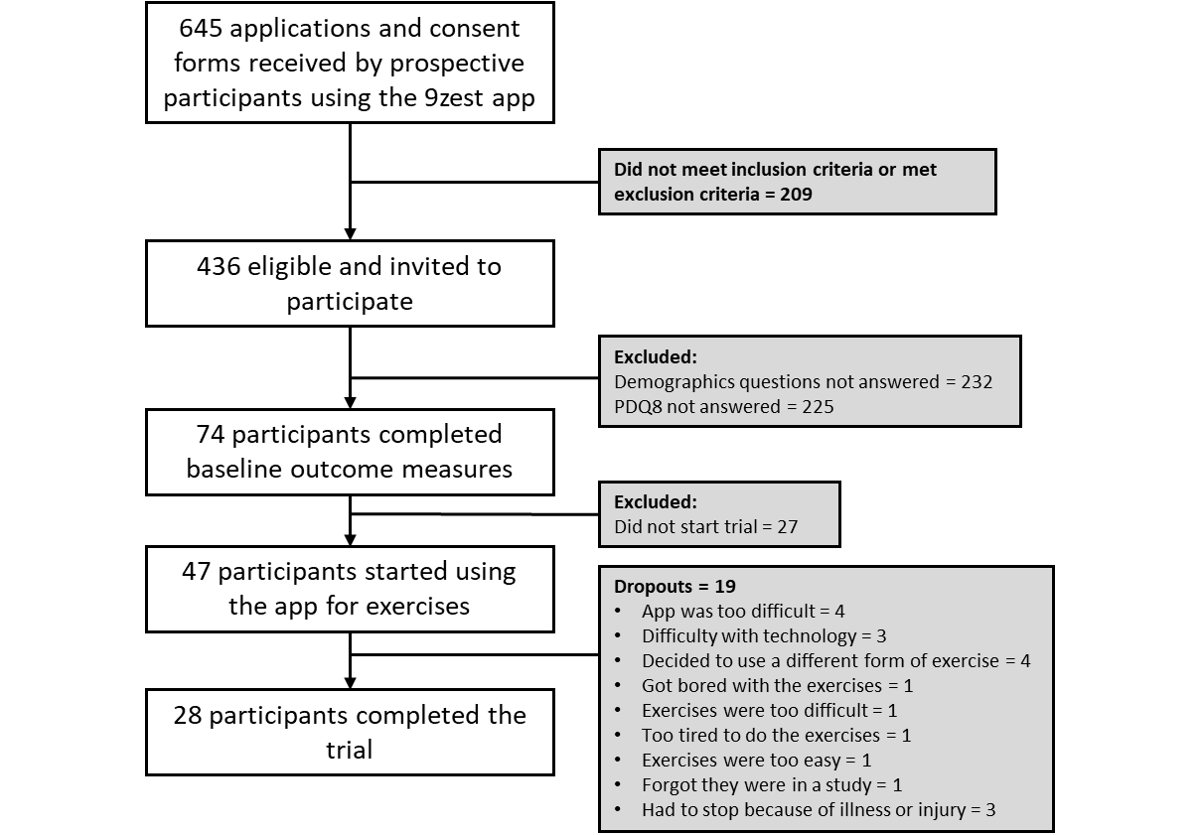
CONSORT flow diagram of study participants.

A total of 28 participants (mean age in years 62.1 [SD 9.6], mean years since diagnosis 3.3 [SD 2.5]; males=6; females=14, and unknown=8) completed the 12 weeks of the study (Figure 6). There were no statistically significant differences between the dropouts and participants that completed the trial for age, sex, and years since diagnosis (Table 1).

**Table 1:** Means, proportions, and statistical comparisons for participant demographics for those who completed the trial and dropouts.

Demographics			Participants who completed the trial		Dropouts		*P* value
N			28		19		NA^a^
Age (years), mean (SD)			62.1 (9.6)		59.7 (8.6)		.42
**Sex**							.13
	Male, n	6		2			
	Female, n	14		17			
	Not reported, n	8		0			
Years since diagnosis, mean (SD)			3.3 (2.5)		4.7 (4.4)		.24

^a^NA: not applicable.

### Aim 1 (Feasibility)

As much as 12 of the 28 participants averaged more than 150 minutes of app usage per week. The remaining 16 participants averaged the following: 120-150 minutes (n=3), 90-120 (n=4), and <90 minutes (n=9). Of the 19 respondents (9 did not respond), a majority felt the app exercise was of value (47% [9/19] strongly agreed, 37% [7/19] agreed, 5% [1/19] neutral, 11% [2/19] strongly disagreed), provided adequate instruction (53% [10/19] strongly agreed, 21% [4/19] agreed, 11% [2/19] neutral, 5% [1/19] disagreed, 11% [2/19] strongly disagreed), was enjoyable (47% [9/19] strongly agreed, 26% [5/19] agreed, 16% [3/19] neutral, 11% [2/19] strongly disagreed), would recommend to other people with Parkinson disease (53% [10/19] strongly agreed, 26% [5/19] agreed, 5% [1/19] neutral, 16% [3/19] strongly disagreed), and was appropriate for my level of function (37% [7/19] strongly agreed, 47% [9/19] agreed, 5% [1/19] disagreed, 11% [2/19] strongly disagreed).

### Aim 2 (Safety)

There were no reported bouts of dizziness, falls, chest pain, or other medical events/issues during the app-guided exercise but there was 1 report of shortness of breath that resolved without the need for medical attention. There were 4 reports of strain/sprain injuries (3 participants: low back pain and 3 episodes of knee pain), none of which necessitated medical attention. Of the 19 respondents (9 did not respond), a majority felt the app exercise was safe (63% [12/19] strongly agree, 21% [4/19] agree, 5% [1/19] neutral, and 11% [2/19] strongly disagree).

### Aim 3 (Signal of Efficacy)

There was a statistically significant improvement for each of the 3 primary endpoints. The STS improved from the premeasurement, mean 11.6 (SD 4.0), to the 8-week measurement, mean 14.3 (SD 4.7; *P*=.01; Hedges g=0.59; 95% CI 0.16-1.04). At the 8-week point, 15/28 (54%) improved beyond the MDC on the STS. The TUG improved from the premeasurement, mean 11.2 (SD 3.9), to the 8-week measurement, mean 8.5 (SD 2.6; *P*<.001; Hedges g=.80; 95% CI 0.46-1.18). At the 8-week point, 8/28 (29%) improved beyond the MDC on the TUG. Lastly, the PDQ8 improved from the premeasurement, mean 6.8 (SD 5.0), to the 8-week measurement, mean 4.1 (SD 5.0; *P*=.01; Hedges g=0.53; 95% CI 0.14-0.94). At the 8-week point, 6/28 (21%) improved beyond the MDC on the PDQ8.

At the conclusion of the 12-week study, 63% (12/19, 9 nonrespondents) felt their condition was better using the Global Rating of Change score. The results of the ANOVAs (premeasurement, 8-week, and 12-week measurements) suggest that there were no additional improvements from the 8-week to the 12-week measurement points for the STS (*P*>.99), TUG (*P*>.99), and PDQ8 (*P*=.94). There were no statistically significant interactions for the factorial ANOVAs to test dosing effect: STS (*P*=.39), TUG (*P*=.41), and PDQ8 (*P*=.86). Likewise, there were no statistically significant correlations for the average time of app exercise usage and change scores on the STS (r=–0.148, *P*=.45), TUG (r=0.113, *P*=.57), and PDQ8 (r=–0.017, *P*=.93).

## Discussion

### Principal Findings

The main purpose of this study was to test the feasibility, safety, and signal of efficacy of the 9zest app for people with Parkinson disease. Results of this study suggest that independent, video-guided exercise using the 9zest mobile app, designed for exercise for people with Parkinson disease, may be safe and feasible with considerable variability in app usage. A majority of participants felt the app-guided exercise was of value and enjoyable, and would recommend it to other people with Parkinson disease. A majority of participants (16/19, 84%) felt the app-guided exercise was not only safe but also appropriate for their current level of function. Despite the variability in app usage, the results suggest a signal of efficacy as there were statistically significant improvements on all 3 outcome measurements (*P*=.01 for STS; *P*<.001 for TUG; and *P*=.01 for PDQ8). Additionally, a majority of participants (12/19, 63%) felt that their condition had improved over the 12 weeks of the study. Based on these promising data, the 9zest app may be a safe, feasible, and useful technology for people with Parkinson disease as an adjuvant to a formalized physical therapy program or for those who wish to exercise independently or who do not have access to a physical therapist or Parkinson disease–specific exercise instruction because they live in a rural or underserved community. However, caution is warranted as larger, well-controlled trials are needed to draw more definitive conclusions. While the study was not designed to make cause and effect inferences, the results suggest an association between exercising via the app and improvement in lower extremity strength (STS), dynamic balance and mobility (TUG), quality of life (PDQ8), and overall improvement (Global Rating of Change). These results are consistent with a beneficial effect of independent exercise using other smartphone apps in other clinical populations [50].

It is important to note that the design of this study was such that participants did not have any contact with members of the research team and all assessments and training were done with in-app programming. This was certainly advantageous from a research resource perspective and it does mimic real-world use of this commercially available app; however, the lack of direct researcher contact/interaction and the requests to carry out assessments may have contributed to the poor on-boarding rate (Figure 6). The major reason for the poor onboarding rate may be that the request to provide demographic information and to perform assessments may have deterred participation as 436 prospective participants expressed interest in participating in the study but only 74 participants provided demographic data and completed outcome measures. This may be due to participant burden or concerns related to privacy (eg, not wanting to share demographic information). Losing 27 after completing assessments (ie, not starting the intervention) may be due to lack of interest or lack of follow through. The lack of interaction with a health care professional or coach may have contributed to the poor on-boarding and retention/adherence rates.

It is noteworthy that only 43% (12/28) of the participants reached the target of at least 150 minutes of app-guided exercise per week. There was considerable variability in usage of the app for exercise. Because the app was used independently with no supervision, it is possible that some participants may have had the app on and running but were not actually performing the exercise along with the video, thereby inflating participation rates. However, due to the study design, there is no way of knowing whether this occurred or not. Additionally, there is no way of knowing if participants “dropped-in” to another exercise program during the 12-week study. Regardless, 2 analyses (mixed factorial ANOVAs and correlational analyses) were conducted to determine if there was a dose effect of app usage on the outcome measures. The results of these analyses did not indicate that there was a dose effect and the very low correlations support this notion despite the fact that other exercise and smartphone app studies have linked exercise exposure to efficacy [51]. Further investigation into the dosing of the 9zest app-guided exercise using a more rigorously controlled design is warranted.

Based on the results of Aim 3, it is clear that the associations of improvement in the 3 main outcomes measures occurred during the first 8 weeks of the study. There were no statistically significant improvements from the 8-week measurement point to the 12-week measurement point for any of the outcome measures. This suggests that the improvement may have plateaued by the 8-week measurement point. However, the Global Rating of Change question, asked at the 12-week measurement point, indicates that a majority felt their condition had improved over the duration of the study.

Behavior change elements integrated into the app may have helped adherence to the app-guided exercise. For instance, incorporating remote Parkinson disease–specific peer coaching [52] or remote supervision by a physical therapist [53] to promote app use may be a helpful way to encourage adherence and promote accountability [52]. While the app currently has several behavior change techniques, including prompts/cues, goal setting, graded tasks, and outcome feedback, to promote exercise that are consistent with other apps for exercise [54,55], these may have been less personalized compared with approaches implemented by someone trained to address behavior change like a physical therapist. By contrast, it is possible that the behavior change elements in the study were ineffective at promoting adherence to the research study or the exercise program. It is also plausible that the app was not engaging enough or lacked the optimized motivational prompts to promote adherence.

### Limitations

The most prominent limitation of the study was the poor onboarding rate entering the study. There was a poor yield from those who were invited to participate (74/436, 16.9%) and a high dropout rate once participants had completed the baseline measurements and started the exercise (19/47, 40%). These challenges are not unique to this study as other app-based studies have also had similar struggles with recruitment/yield and dropout [56]. As much as 7 of the 19 dropouts had problems with the app or technology, which may be an inherent app-related problem or simply a challenge for older adults using unfamiliar technology. This suggests that some human interaction (eg, a remote health care professional/coach) in studies like this, especially in the early stages of research, may be important design elements to promote better onboarding and adherence. The poor yield and high dropout rate resulted in a considerably smaller sample size than was estimated and was also smaller than the a priori sample size estimate. The study participants were also a sample of convenience which may have resulted in a biased sample of those comfortable with the technology and those who were already motivated to exercise. While the sample size was sufficient for the primary endpoints of the study, it was likely underpowered for the Aim 3 factorial ANOVAs. It is also important to note that low levels of app use do not necessarily equate to actual exercise levels as it is possible that participants were exercising without the app. Another limitation of the study was the remote testing of performance-based measures such as the STS and TUG, which have not been psychometrically vetted using a remote test. Two participants strongly disagreed that the app was safe. In fact, these same 2 participants strongly disagreed on every Likert question despite both being regular users of the app and both improving over the course of the study. There were no consistent characteristics, patterns, or themes in these participants’ data. It is possible that they may have misinterpreted the direction of the scale. Lastly, adverse events were only asked every 2 weeks and this length of time may have increased the chance for recall bias (ie, under- or over-reporting because of poor memory).

### Conclusion

For those participants who completed this study, independent, video-guided exercise using the 9zest mobile exercise app for people with Parkinson disease was safe and feasible and a majority of participants felt the app-guided exercise was enjoyable, provided adequate instruction, and would recommend it to others. While this study was not designed to determine cause and effect, the results provide evidence for a signal of efficacy as there were improvements in lower extremity functional strength, mobility and dynamic balance, and quality of life after 8 weeks of participation which were sustained at 12 weeks. The poor onboarding and adherence may suggest a limited generalizability only to those that are able to interact successfully with the technology.

## Abbreviations

MDC: minimal detectable change
PDQ8: Parkinson’s Disease Questionnaire 8
STS: 30-second Sit-To-Stand
TUG: Timed Up and Go
